# Quantifying Agreement between Anatomical and Functional Interhemispheric Correspondences in the Resting Brain

**DOI:** 10.1371/journal.pone.0048847

**Published:** 2012-11-08

**Authors:** Hang Joon Jo, Ziad S. Saad, Stephen J. Gotts, Alex Martin, Robert W. Cox

**Affiliations:** 1 Scientific and Statistical Computing Core, National Institute of Mental Health, National Institutes of Health, Bethesda, Maryland, United States of America; 2 Cognitive Neuropsychology Section, Laboratory of Brain and Cognition, National Institute of Mental Health, National Institutes of Health, Bethesda, Maryland, United States of America; University of Montreal, Canada

## Abstract

The human brain is composed of two broadly symmetric cerebral hemispheres, with an abundance of reciprocal anatomical connections between homotopic locations. However, to date, studies of hemispheric symmetries have not identified correspondency precisely due to variable cortical folding patterns. Here we present a method to establish accurate correspondency using position on the unfolded cortical surface relative to gyral and sulcal landmarks. The landmark method is shown to outperform the method of reversing standard volume coordinates, and it is used to quantify the functional symmetry in resting fMRI data throughout the cortex. Resting brain activity was found to be maximally correlated with locations less than 1 cm away on the cortical surface from the corresponding anatomical location in nearly half of the cortex. While select locations exhibited asymmetric patterns, precise symmetric relationships were found to be the norm, with fine-grained symmetric functional maps demonstrated in motor, occipital, and inferior frontal cortex.

## Introduction

The human brain is composed of two grossly symmetric cerebral hemispheres that are interconnected by white matter tracts, mainly through the corpus callosum, as well as through the anterior and posterior commissures. At a large spatial scale, the pattern of cortical folding in terms of gyri and sulci appears largely symmetric. This symmetry is echoed at the more detailed scale of cell-to-cell synaptic connections and cortical maps, with anatomical studies in animals revealing an abundance of symmetric connections across corresponding locations in the two hemispheres [1], [2], [3]. In contrast to this seeming symmetric anatomical organization, there are striking and well-known examples of cognitive functions in humans that are strongly lateralized to one hemisphere, such as language to the left hemisphere and visuospatial and attentional abilities that are more lateralized to the right hemisphere [4], [5], [6], [7]. These joint observations of cortical symmetry and lateralization pose a basic puzzle for our understanding of large-scale brain organization: how does functional lateralization emerge in the context of gross anatomical symmetry?

In humans, it is not possible to directly measure anatomical connections through the standard anatomical tracer techniques that are used in animals. Instead, researchers commonly use noninvasive methods such as magnetic resonance imaging (MRI) to assess structural and functional relationships between pairs of brain locations, using techniques such as diffusion tensor imaging (DTI) [8], [9] to estimate the presence of white-matter tracts, and the blood-oxygen-level-dependent (BOLD) functional MRI (fMRI) to estimate functional correlations among task-evoked or resting voxel timeseries [10], [11], [12], [13]. Indeed, the last decade has seen an explosion of studies that measure patterns of temporally covarying BOLD activity while subjects are at rest in order to assess functional relationships in large-scale brain networks [12], [14], [15], [16], [17], [18], [19], . One of the most common observations is that the timeseries from a seed region-of-interest (ROI) in one hemisphere is strongly correlated with the timeseries in the corresponding location in the opposite hemisphere in humans [22], [23], [24] and in monkeys [25], [26], [27]. In the first such study, Biswal et al. observed that a seed timeseries extracted from the left motor cortex was highly correlated with the same relative location in the right motor cortex during rest, as well as with other motor-related regions (e.g. supplementary motor area), that were identified during a separate finger-tapping localizer scan [13], [14]. This finding has been replicated many times in motor cortex as well as in a variety of other brain regions [10], [21], [25]. More recent studies have attempted to characterize the generality of these cross-hemispheric correlations over the whole brain [28], [29], [30], as well as to clarify the spatial precision of the cross-hemispheric maps in somatotopic cortex [31]. Studies evaluating the complementary issue of cross-hemispheric asymmetries have also been carried out [32], [33], [34], [35]. However, one major difficulty facing all of these studies is that it is difficult to be precise about exactly which two points are corresponding in the two hemispheres. Studies that have attempted to quantify symmetries/asymmetries have simply “flipped” the *x*-coordinate value within standard anatomical space, assuming that this estimate will be adequate. However, this approach is at the mercy of what may be quite striking variations in the detailed gyral folding patterns in the two hemispheres, some of which may be systematic across subjects, as well as differences in overall brain shape from subject to subject and other anatomical differences [36], [37].

A better solution is to utilize a surface-based representation of the cortical surface, aligned across subjects [38], [39], [40], [41], [42]. Correspondence between the two hemispheres can then be estimated relative to cortical landmarks in the two hemispheres. In the current paper, anatomically corresponding locations are defined relative to a large number of gyral and sulcal landmarks, allowing precise estimates of homotopic locations throughout the entire cortex. We first identify corresponding locations throughout both hemispheres and quantify the improved precision over the standard flipping method. We then quantify the agreement of anatomical correspondences with the physiological or “functional” correspondences, defined here as the peak value of the cross-hemispheric resting-state (RS) correlation maps. Finally, we examine the fine-grained nature of the cross-hemispheric correlation maps in well-known cortical maps, such as somatotopic and retinotopic cortex, as well as in areas without known maps.

## Materials and Methods

### Subjects

Forty male, right-handed, physically healthy volunteers (mean age 19±2.5 years), without a history of known psychiatric or neurological disorders, participated in our experiment. All had normal or corrected-to-normal vision. Written informed assent and consent were obtained from all participants and/or their parent/guardian. The National Institutes of Health Institutional Review Board approved the study.

### MRI Acquisition

During MRI scan sessions, subjects were instructed to clear their minds and fixate on a black cross in the center of the screen. All MR image data were collected using a GE 3T scanner with 8-channel head coil array. RS-fMRI time series were acquired using a T2*-weighted gradient echo pulse sequence with high spatial resolution (1.7×1.7×3.0 mm^3^, TR = 3.5 s, TE = 27 ms, flip angle = 90°) accelerated by the SENSE method. Each of the RS-fMRI scans lasted for 490 s or 140 volumes. EPI data passed the sudden motion detection of AFNI program ‘afni_proc.py’ at the threshold level 0.3 mm for Euclidean L2 norm of motion displacement during each TR interval (see also Power et al., 2012). A respiration belt was used to measure respiration volume and a pulse oximeter to monitor heart rhythm. Belt diameter and pulse oximeter readings were sampled at 50 Hz, with recording onset triggered by the scanner’s slice-selection trigger pulse. For each subject, an MPRAGE sequence was used to acquire two high-resolution (0.9×0.9×1.2 mm^3^) T1-weighted anatomical images.

### Preprocessing and De-noising

AFNI cross-modal registration software was used to align anatomical images for each subject to the fifth volume of the RS-EPI time series [43]. Aligned anatomical images were then processed with FreeSurfer’s automated pipeline for generating cortical surface models and whole brain segmentation [44]. The following procedures were carried out using AFNI’s suite of programs [45], [46]. Individual cortical surfaces were geometrically corrected, standardized (36,002 nodes per hemisphere), and then registered to the cortical surfaces of the N27 template brain [47], to ensure that the N27 template corresponds with individual cortical surfaces [39], [40]. Preprocessing of the RS-EPI time series was carried out using the basic ANATICOR method, which uses regression to remove recorded nuisance physiological signals such as cardiac and respiratory signals, as well as head coil/hardware artifacts [48]. Additional 1-TR delayed regressors of the nuisance variables were also included in order to remove any additional delayed effects of noise sources. Overall data processing procedures were described in a diagram in the supporting information (see Figure S1).

### Landmark-Based Correspondence

**Figure 1:**
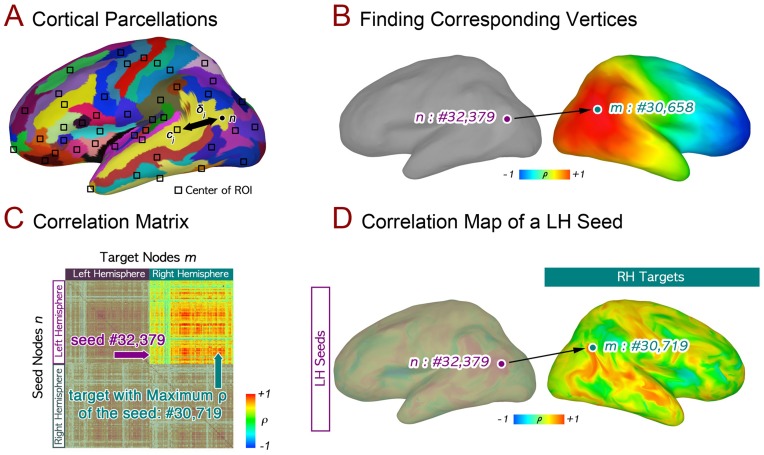
Anatomical and functional correspondences across hemispheres. A high-dimensional coordinate system is introduced to find the anatomical correspondence (or “anatomical correspondence”) of a seed node in the opposite hemisphere. (**A**) Cortical parcellations and calculation of labeling vectors to represent the location of a cortical node from the centers of FreeSurfer regions-of-interest (ROIs). (**B**) The anatomical correspondence is the node that has the most similar labeling vector to that of each seed. (**C**) Correlations between the resting timeseries of all pairwise vertex combinations, averaged across all subjects (seed nodes along the y-axis, target nodes along the *x*-axis). One seed n (node #32,379 in the LH) in the figure is correlated with multiple target nodes, shown using color along its corresponding row (the 32,379^th^ row in the matrix; see colorbar). (**D**) The “functional correspondence” m (node #30,719 in the RH) of the seed n is defined as the RH target node with time series maximally correlated to the data from n.

While the number of nodes in the standardized left and right hemisphere surfaces is the same, identical node indices in the two hemispheres do not represent anatomically homologous locations. Corresponding cortical nodes in the two hemispheres were identified by finding nodes with the most similar pattern of relative distances from within-hemisphere gyral/sulcal landmarks. We assigned to each cortical node a 74-dimensional label vector containing the distances along the surface (geodesic) from that node to the centroid of each of the 74 parcellated cortical regions provided by FreeSurfer, based on gyral boundaries, to redefine locations on the cortical surfaces instead of the Cartesian coordinate (*x, y, z*) as showed in Figure 1A:

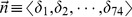
(1)where 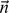 is the label vector to define the position of a node in the hemisphere; *δ_j_* is the geodesic distance along the cortical surface between the node and the centroid of *j*-th FreeSurfer cortical region (i.e. the length of the shortest path along the surface between the points), which is calculated on the standardized smooth white matter surfaces by SUMA’s *SurfDist* program. The “anatomical” correspondence of a cortical node can be determined by finding the node in the opposite hemisphere whose label vector has the largest Pearson correlation (ρ) with the seed node’s label vector. Therefore, the anatomical correspondence of a node is the node on the contralateral hemisphere with the most similar label vector as shown in Figure 1B.

### Functional Correspondence

In a typical RS functional connectivity analysis, a seed’s time series from a particular location is correlated with the remaining time series in the brain [13]. Correlations between the seed and other locations are used as an indication of functional connectedness, which can be represented as a cross-correlation matrix among the time series of every node in a brain model. In order to avoid aliasing of cerebrospinal fluid and white matter signals into the grey matter signal estimates, the residual time series from the regression model were mapped onto cortical surfaces using an average kernel with ten sampling points evenly distributed along a line centered between smooth white matter and pial surfaces and extending 80% of the thickness between corresponding nodes on the two surfaces. The mapped time series were smoothed with a heat kernel that resulted in 8 mm full-width-at-half-maximum noise spatial correlation structure on the white matter surface [49], [50], [51], [52]. For each subject, we calculated the full cross-correlation matrix for the time series of every node on both hemispheres, which is shown in Figure 1C. These individual correlation matrices were then averaged to make a group correlation matrix reflecting common characteristics of connectivity. For correlation analysis, each node served as both a seed and target location. For determining functional connectivity across hemispheres, a node in one hemisphere is the seed, and all nodes in the contralateral hemisphere are its targets as illustrated in Figure 1D. Node colors in the RH target show correlation coefficients (ρ) with a seed *n* of the LH. The functional correspondence (FC) of LH seed *n* is defined here as the contralateral target node whose time series is maximally correlated to that of the seed (node *m* in Figure 1D). It is important to note that the term functional “correspondence” is used more loosely here than for the anatomical correspondence (AC), as there is no in-principle constraint that the FC be near the AC. Indeed, it could be located far away from the homotopic cortical position of the seed node.

### Quantification of Functional Correspondence Across Hemispheres

**Figure 2:**
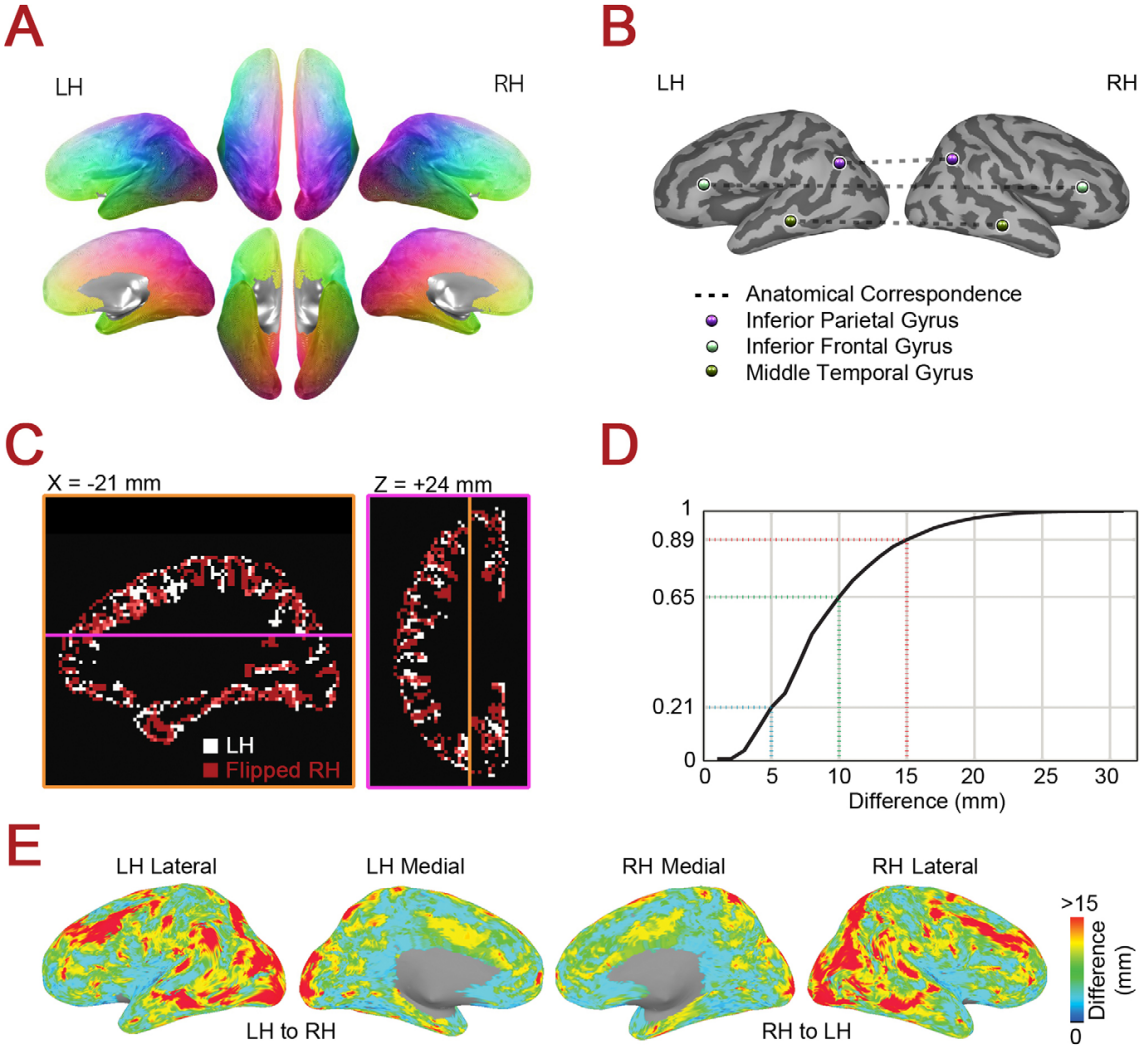
Anatomical correspondences using cortical landmarks versus coordinate flipping. (**A**) Anatomical correspondences across hemispheres found using gyral and sulcal landmarks. Lateral/dorsal views are shown in the top row, and medial/ventral views are shown in the bottom row. Anatomical correspondences in the two hemispheres are given the same unique color. (**B**) Example anatomical correspondence mappings are shown for three vertices in the inferior frontal gyrus, the middle temporal gyrus, and the inferior parietal cortex, demonstrating the proper function of the landmark-based method. (**C**) Mismatches between left and *x*-coordinate “flipped” right grey matter ribbons for the TT_N27 brain template (Talairach coordinates). (**D**) Cumulative histogram for the Euclidean distances calculated in the volume between landmark-based anatomical correspondences and the locations found by flipping the *x*-coordinate (over all grey matter voxels). (**E**) Surface cortical projection of the voxel values contributing to (D), with distances on the left versus right of the figure found using the left versus the right grey matter ribbons, respectively.

**Figure 3:**
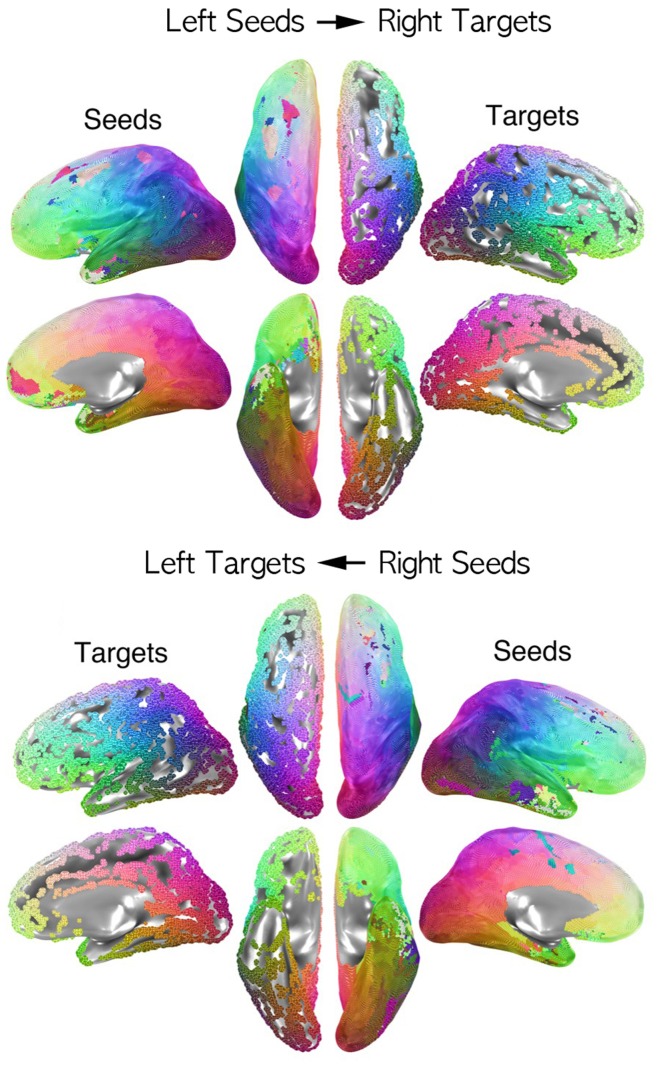
Functional correspondences by seed hemisphere. The functional correspondences on the right hemisphere (RH) of left seed points are presented in the upper figure. Colors of a node in the LH correspond to the anatomical color from the “targets” side (using color scheme in Figure 1A). Uncolored target vertices in the RH are locations that were not maximally correlated with any seed vertex in the LH. RH seed points and their functional correspondences are presented in the lower half of the figure. Most vertices in both hemispheres are maximally correlated with locations close to their corresponding anatomical correspondences.

**Figure 4:**
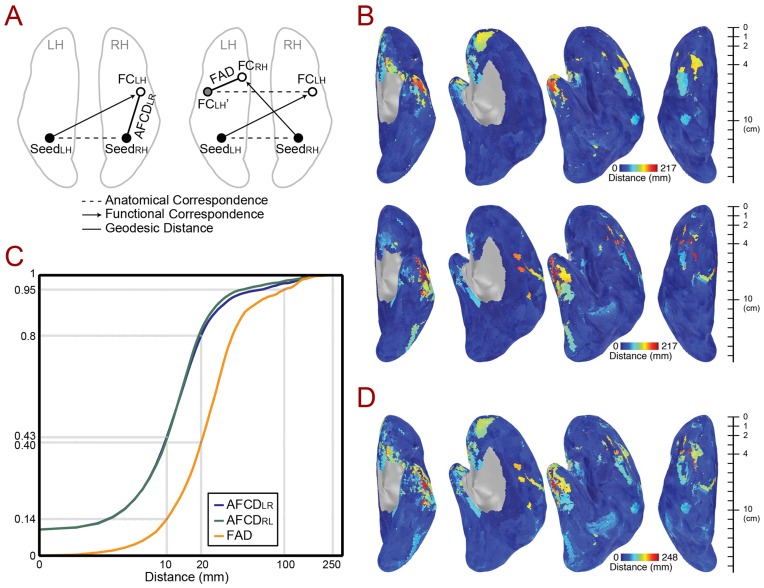
Quantification of functional correspondency and asymmetry. (**A**) Anatomy-to-functional correspondence distance AFCD_LR_ for a left seed point Seed_LH_ (black circle in the left hemisphere) is defined as the surface geodesic distance from the anatomical correspondence (black circle in the right hemisphere) and the functional correspondence FC_LH_ (open circle in the right hemisphere) of Seed_LH_. AFCD_RL_ for a right seed point Seed_RH_ can be determined in the left hemisphere in the same manner. The functional asymmetry distance (FAD), shown to the right, describes the geodesic distance between two functional correspondences that result from anatomical correspondences (e.g. Seed_LH_ and Seed_RH_), when the functional correspondences have been remapped to the same hemisphere (e.g. FC_LH'_ and FC_RH_). (**B**) AFCD for left and right seeds are presented in the upper and lower rows, respectively, using color (see colorbars). The scale bars to the right are the approximated scales for distance comparison (in cm). (**C**) Cumulative histograms of AFCD (left versus right seeds) and FAD, calculated over all vertices. (**D**) FAD values for all vertices presented using color (see colorbar). Small FAD values indicate symmetric patterns of functional correspondences across the hemispheres whereas high values indicate asymmetric patterns.

Each seed node gets both anatomical correspondences and functional correspondences on the contralateral hemispheres illustrated in Figure 2A and Figure 3. We define the geodesic distance between anatomical and functional correspondences as the Anatomy-to-Functional Correspondence Distance (AFCD) (see Figure 4A). A small AFCD indicates that RS fluctuations at one anatomical location have their maximal correlation near the corresponding location in the contralateral hemisphere. The AFCDs are computed for all seed nodes of both hemispheres. In principle, large values of AFCD do not necessarily indicate an asymmetric pattern across the two hemispheres, since two anatomical correspondences can yield distant functional correspondences that are anatomical correspondences of each other. The symmetric or asymmetric nature of the patterns can be described by one quantity called the Functional Asymmetry Distance (FAD), defined as the distance between the functional correspondences of 2 anatomically corresponding points when projected to same hemisphere through the anatomical mapping (right side of Figure 4A).

### Assessing Discrepancies between Landmark-Based Correspondence and Coordinate Flipping

Given that the more standard method of determining corresponding points in the two hemispheres involves the simple operation of “flipping” (i.e. reversing) the sign of the *x*-coordinate in standard anatomical space, it is important to estimate the relative benefit of the landmark-based method. In order to quantify this benefit, maps of the anatomical correspondences determined through the landmark-based method were converted back to volume space using the mapping of the N27 surface template to its corresponding Talairach template in the volume (TT_N27 brain). For each grey matter seed voxel in the TT_N27 brain, the Euclidean distance in volume space was then calculated between the volume-converted anatomical correspondence in the opposite hemisphere and the correspondence expected by flipping of the *x*-coordinate (as in Figure 2D). This set of distances for the TT_N27 brain could then be graphically rendered either in the volume or back on the N27 surface template for ease of viewing (Figure 2E).

## Results

### Anatomical Correspondences through Cortical Landmarks Versus Coordinate Flipping

Using the landmark-based correspondence method for cortical surfaces (shown graphically in Figure 1), we determined anatomically corresponding cortical locations between the hemispheres. Correspondences were found by comparing distances on the cortical surface between each vertex and the centroids of automatically segmented regions of interest from FreeSurfer from the same hemisphere [44]. Pairs of vertices in the two hemispheres with the most similar pattern of relative distances are defined here as “anatomical correspondences”. In Figure 2A, each vertex in the left hemisphere is shown with a unique color, and its anatomical correspondence in the right hemisphere is given the same color. Note the nicely symmetric color map across the two hemispheres, indicating a well-ordered relationship. Figure 2B shows 3 single vertices in the left hemisphere sampled from the left inferior frontal gyrus, the left inferior parietal cortex, and the left middle temporal gyrus, along with their anatomical correspondences in the right hemisphere. Note the good agreement of the left and right hemisphere points relative to the gyral/sulcal landmarks in each case, shown on the inflated cortical surface in two shades of gray (dark gray = sulcus, light gray = gyrus). Thus, at the scope of the whole-brain, the landmark-based correspondence method operates as expected, with single points paired at the same relative position to gyral/sulcal boundaries.

Given the prevalence of the coordinate flipping method and its ease of implementation, it is important to quantify the relative benefit of finding correspondence through cortical landmarks. In order to evaluate this, the surface-based representations of the anatomical correspondences were translated back into standard volume space for the TT_N27 brain [47], and the Euclidean distance was calculated between the anatomical correspondence of each gray matter voxel and its correspondence calculated through flipping the *x*-coordinate value (i.e. multiplying by -1). Given the poor spatial agreement of the grey matter ribbon in the two hemispheres (shown in Figure 2C), it is perhaps not surprising that the discrepancies between the two methods can be quite large, with nearly 80% of anatomical correspondences residing more than 5 mm away from the expected flipping locations (summarized over voxels in both hemispheres in the histogram in Figure 2D). Indeed, flipping can commonly lead to estimated correspondences that are outside grey matter, either in white matter of the opposite hemisphere or outside the brain. For ease of viewing, the distances between the two methods in the volume have been rendered back onto the surface in Figure 2E. Problem spots for the flipping method (in which the discrepancies are 15 mm or more, shown in red) are notable in the lateral frontal, temporal, and occipital cortices, where cortical folding can be somewhat irregular and idiosyncratic for a given subject. The benefits of finding hemispheric correspondence through cortical landmarks are particularly clear in these locations, although measurable benefits are present throughout the cortex. Since extensive spatial smoothing is often applied in volume-based coordinate flipping studies in order to improve interhemispheric correspondences, a further benefit of the surface-based landmark approach is that it may permit a reduction in the extent of spatial smoothing necessary for good correspondence, thereby enhancing the effective spatial resolution of the data.

### Quantifying Symmetry between Anatomical and Functional Correspondences Across Hemispheres

After finding anatomical correspondences for the two hemispheres, we then determined the functional correspondences across hemispheres by finding the vertex exhibiting the maximal cross-hemispheric correlation for each seed vertex (Figure 3). Throughout most of the cortex, functional correspondences are near their anatomical correspondences in the contralateral hemisphere, indicated by a similar color layout for seed and target hemispheres. Neighboring seed nodes occasionally share the same precise functional correspondence in the contralateral hemisphere, leaving some target nodes without any corresponding seed (e.g. note the territories of the cingulate sulcus and posterior superior temporal sulcus in Figure 3). Discrepant colors in the seed maps of Figure 3 indicate that the functional correspondence in the contralateral target hemisphere is not near the anatomical correspondence, with the precise color indicating the position of the functional correspondence in the target hemisphere (target color maps match those for the same vertex positions in Figure 2A).

The distance along the cortical surface between the anatomical and functional correspondences shown in Figures 2 and 3 is defined as the Anatomy-to-Functional-Correspondence Distance (AFCD) (graphically defined on the left side of Figure 4A). AFCD (in mm) for each vertex is shown using color in Figure 4B (top: left seeds, bottom: right seeds), with corresponding histograms shown in Figure 4C. Perhaps the most notable finding is that the majority of the brain has relatively small AFCDs, shown in blue colors, with 80% of vertices having a distance of less than 2 cm between anatomical and functional correspondences, indicating cross-hemispheric symmetry in resting-state correlation maps. Note that this distance should *not* be compared to the Euclidean distances shown in Figure 2D, since AFCD is a measure of the spatial correspondence between an anatomical and a physiological quantity rather than a measure of the mismatch of two anatomical coordinate systems. Distances calculated along the contour of the cortical surface will also tend to be larger than those calculated in the volume, since the latter ignore the folding in the cortical surface. In a few select locations (e.g. the lateral anterior temporal cortex), the AFCDs can be much larger, yielding distances of more than 21 cm along the surface (shown in red). However, large values of AFCD do not necessarily indicate an asymmetric pattern across the two hemispheres. This is because two seed vertices that are anatomical correspondences of one another might yield functional correspondences that are far away from the seed locations, yet are themselves anatomical correspondences (i.e. they fall at anatomically symmetrical positions, forming a “criss-cross” pattern when viewed from above). In order to address this issue, the Functional Asymmetry Distance (FAD) instead calculates the discrepancy between resulting functional correspondences, using the map of anatomical correspondences to project both functional correspondences to the same hemisphere (see right side of Figure 4A). As can be seen from Figure 4D, the FAD values look very similar to the AFCD values in Figure 4B, suggesting vertices with high values of AFCD do indeed correspond to asymmetric patterns.

**Figure 5:**
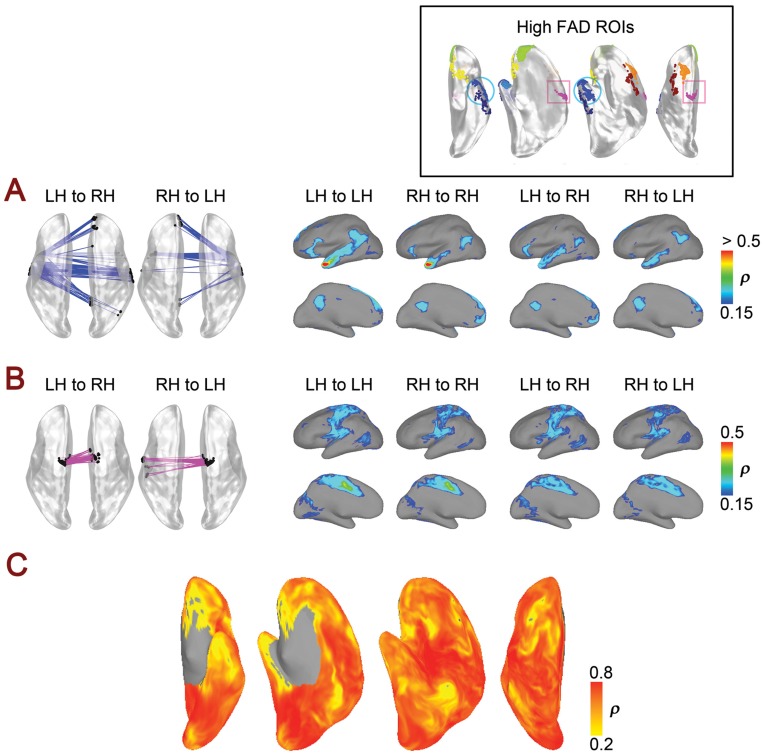
Correlation patterns of regions with high asymmetry. The seed regions with the highest FAD values (>100 mm ∼ upper 5%) are shown in the right upper box. Small clusters with fewer than 20 vertices are excluded. Correlation patterns are illustrated in **A** and **B** for two example ROIs. (**A**) Cross-hemispheric correlation patterns for the temporal pole/middle temporal gyrus ROI (indicated by the light blue circle in the upper box), shown to the left with the dorsal view. Lines connect individual seed vertices to their functional correspondences. The lateral and medial views in the right illustrate the averaged within- and across-hemisphere correlation maps for all vertices in the ROI. (**B**) Cross-hemispheric correlation patterns for the supplementray motor area ROI (indicated by the magenta square in the upper box; presented as in **A**). (**C**) Similarity between whole-hemisphere contralateral correlation maps that were elicited by each pair of anatomical correspondences. Pairs of vertices yielding a positive correlation of ρ >0.2 are shown using color (see the colorbar).

The potentially asymmetric functional correspondence patterns detected using the FAD were then investigated in more detail by organizing them into separate clusters of contiguous vertices with similar values of FAD (see ROI key in Figure 5). For the clustering, the seed regions were thresholded above FAD = 100 mm (upper 5% of values), excluding small clusters with fewer than 20 vertices (∼ 50 mm^2^ area). Maps of the functional correspondences for two example clusters in the lateral anterior temporal cortex and the supplementary motor cortex are shown in Figure 5A and 5B, respectively. In Figure 5A, seeds in the left anterior temporal cortex have most of their functional correspondences in the symmetric locations in the right hemisphere, as well as in the posterior cingulate and medial prefrontal cortex. In contrast, seeds in the right anterior temporal lobe have relatively more functional correspondences in the left medial frontal cortex, with a good number still residing in the left anterior temporal cortex and a few in the left posterior cingulate. These various locations constitute language- and social-related areas [7], [53], [54] and/or subcomponents of the well-studied “default” network [12], [20]. In order to gain a more complete picture of the patterns of correlation for these vertices, we also calculated the average correlation map for these seed locations within and across hemispheres rather than simply relying on the location of maximum correlation, shown to the right of Figure 5A. Rather than serving as an example of a pronounced asymmetric pattern of correlation, the average correlation maps suggest quantitative and not qualitative differences in the maps between hemispheres, with the position of maximum cross-hemispheric correlation occurring in one of the other areas making up the large-scale brain network. A similar example is shown in Figure 5B, but for supplementary (medial) motor and primary (lateral) motor areas, with the right hemisphere seeds eliciting functional correspondences in the left lateral motor cortex (see Figures S2 and S3 for the remaining ROIs). In both cases, the average correlation maps appear much more symmetrical than the functional correspondences (based on the maximum correlation) would indicate. Consistent with this, Figure 5C shows that the graded cross-hemispheric correlation map elicited by each vertex and its anatomical correspondence in the other hemisphere are themselves positively correlated with one another throughout the cortex, even for vertices with high FAD. Differences in the temporal signal-to-noise ratios (tSNR) across hemispheres were assessed, but they failed to account for the locations of high FAD (see Figure S4).

### Functional Correspondences can be Organized into Precise and Symmetric Spatial Maps

**Figure 6:**
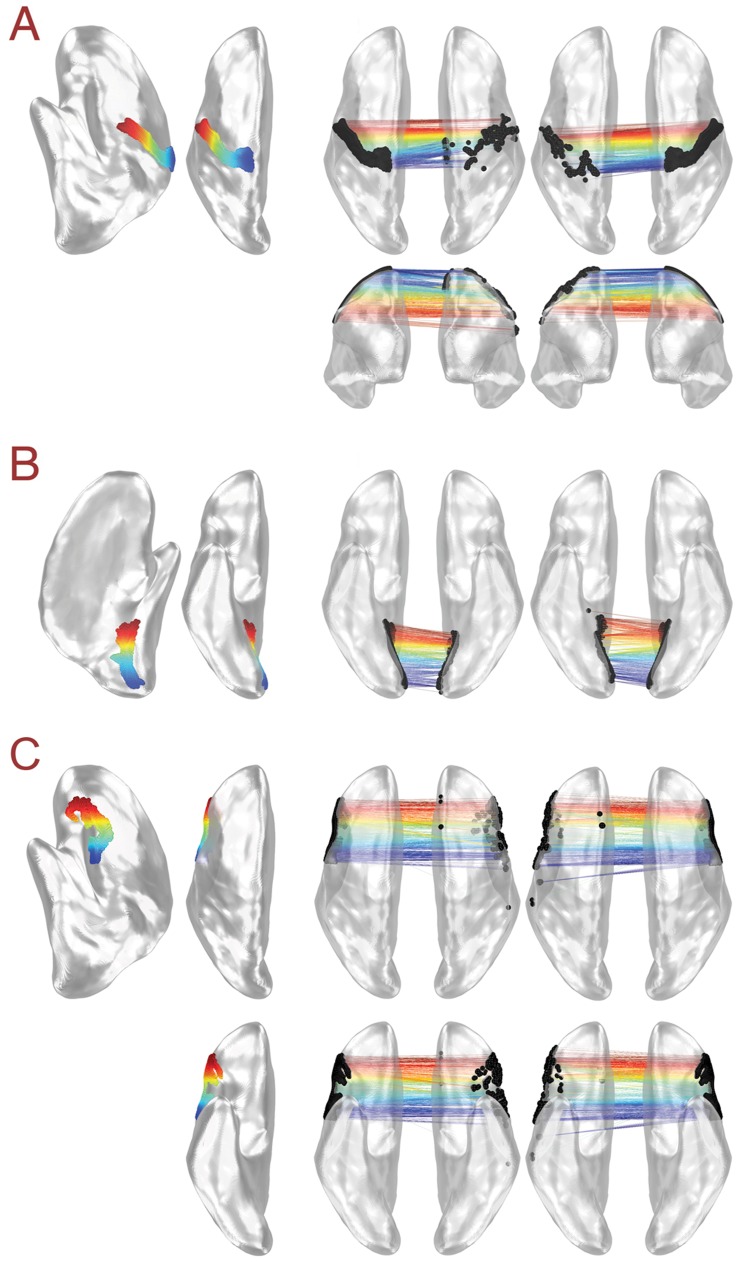
Fine-grained functional symmetry in somatotopic and retinotopic cortex. (**A**) Seed points from somatotopic cortex in the central sulcus are shown on the left hemisphere in lateral and dorsal views. Seed vertices are arranged on the cortical surface from ventral (red) to dorsal (blue) using color. Dorsal and anterior views of the functional correspondences resulting from left versus right hemisphere seed vertices are shown in the middle and right columns, with position along the central sulcus indicated by the colors of the connecting lines. (**B**) Seed points from retinotopic cortex in the calcarine sulcus are presented on the left hemisphere in medial and ventral views. Seed vertices are arranged along the calcarine sulcus from anterior (red), which represents more peripheral aspects of the visual field, to posterior (blue), representing more foveal aspects. Ventral views of the functional correspondences resulting from left versus right hemisphere seed vertices are shown in the middle and right columns, with position along the calcarine sulcus indicated by the colors of the connecting lines. (**C**) Seed points from the inferior frontal gyrus are presented on the left hemisphere in lateral, dorsal, and ventral views. Seed vertices are arranged along the inferior frontal gyrus from anterior (red) to posterior (blue) using color. Dorsal and ventral views of the functional correspondences resulting from left versus right hemisphere seed vertices are shown in the middle and right columns, with position along the inferior frontal gyrus indicated by the colors of the connecting lines.

While some vertices exhibit high AFCD and FAD values, in which the maximum correlation and the average correlation can yield different pictures about the symmetric organization of cross-hemispheric correlation maps, the majority of vertices yield low AFCD/FAD values. This suggested that precise maps of the maximum cross-hemispheric correlation (i.e. functional correspondence) may exist throughout much of the cortex. We examined the fine-grained nature of functional correspondence maps in two regions of cortex with well-known functional maps, somatosensory/motor cortex (in the territory of the central sulcus) and early visual cortex (in the territory of the calcarine sulcus), as well as in an area with no known fine-grained map, the inferior frontal gyrus. Figure 6, using color to represent spatial position, shows that precise, symmetric functional correspondence maps are present in all three brain regions. A variety of the other FreeSurfer ROIs used to calculate anatomical correspondences also exhibit low average FAD values (see Table S1 for all ROIs used). Taken together, these results indicate the widespread presence of fine-grained cross-hemispheric correlation maps. They illustrate the utility of the maximum correlation value in viewing cross-hemispheric relationships, as well as finding corresponding anatomical locations through cortical landmarks.

## Discussion

In the current paper, we have presented a method for finding corresponding locations in the two cerebral hemispheres using gyral/sulcal landmarks. When examining single vertices on the cortical surface, this approach correctly identifies anatomical correspondences in the other hemisphere at the same relative position to the gyri and sulci (e.g. inferior frontal gyrus). In contrast, the standard method of coordinate flipping commonly identifies positions outside of grey matter and/or the corresponding gyrus/sulcus. Using the landmark-based method and two distance metrics on the cortical surface (AFCD and FAD), we systematically characterized the agreement of the position of maximum correlation in the other hemisphere during rest (i.e. the functional correspondence) and the anatomical correspondence, as well as the degree of symmetry in these patterns across the hemispheres. These patterns were found to be largely symmetric. For the few vertices showing asymmetric patterns using the maximum correlation, the full correlation patterns across the hemispheres were still largely symmetric, indicating quantitative rather than qualitative functional asymmetries. The advantages of using the maximum correlation were demonstrated clearly by the ability to characterize fine-grained cross-hemispheric cortical maps amongst functional correspondences in areas with well-known maps (e.g. motor and occipital cortex), as well as in a brain region with no previously described fine-grained map (e.g. inferior frontal gyrus). These quantitative and sometimes subtle relationships would be difficult to investigate using the standard method of coordinate flipping, highlighting the strengths of finding correspondences through cortical landmarks.

One clear implication of the widespread observations of point-to-point symmetry across the hemispheres in the resting-state correlations is that artifactual explanations such as hardware, motion, and non-neural physiological artifacts become progressively unlikely. Furthermore, explanations in terms of explicit cognitive processes and general mental state will have a difficult time addressing the full spatial extent of these results without assuming that each subject is mentally rehearsing their full sensory, cognitive, and motor repertoires every few minutes (i.e. during a single fMRI run). A much more likely explanation is that these results are driven by internally generated, coordinated fluctuations in neurally derived components of the BOLD signal, reflecting a combination of mono- and polysynaptic interactions [55], [56]. While explicit cognitive processes can indeed have a measureable, albeit modest, effect on resting-state correlations within the “default” network [57], [58], the current results complement the findings of other recent studies of spontaneous BOLD fluctuations in anesthetized monkeys [59], [60] and sleeping humans [61], [62] in ruling out the exclusive contribution of explicit cognition. However, the extent to which the symmetric correlation patterns that we observe are carried by direct projections via the corpus callosum is unclear. A recent resting-state study of patients with complete agenesis of the corpus callosum (AgCC) reported largely identical cross-hemispheric correlation patterns to normal controls over a wide set of brain regions [63]. Whether or not AgCC patients would exhibit the same sorts of fine-grained symmetric functional maps reported here is unknown. It is also noteworthy that homotopic callosal connections in early visual areas in monkeys are primarily restricted to representations of the vertical meridian [64], [65], leaving much of visually responsive cortex without callosal inputs. Visual cortex in the depths of the calcarine sulcus in humans, shown here to exhibit fine-grained functional correspondency, is more involved in representing the horizontal meridian [66], [67], potentially casting further doubt on the role of the callosum in mediating all of these functional relationships. Nevertheless, some aspects of our results are indeed reminiscent of callosal anatomical connections in monkeys. For example, both owl and macaque monkeys lack callosal connections in the territories of the posterior superior temporal sulcus and the inferior lateral temporal cortex [68], [69], similar to the target locations in Figure 3 that correspond to no seeds. We would suggest that these similar patterns of contralateral connectedness in different species and modalities are not a coincidence and might reflect the formation of direct callosal connections over fine spatial scales.

The striking patterns of symmetry present in the current study might be taken as inconsistent with the lateralization of cognitive functions such as language and visuospatial attention. How is it that these phenomena can co-exist? The first point to make is that cross-hemispheric symmetry versus asymmetry does not necessarily equate to a bilateral versus unilateral functional organization. For example, if the left inferior frontal gyrus is more involved in language processing than the right inferior frontal gyrus, it does not logically require the right inferior frontal gyrus to be “weaker” in its respective cortical interactions. Instead, the right inferior frontal gyrus could be engaged at an equal level in some other cognitive function. The relevant distinction is in what information processing content the brain regions carries, and this information may not be readily apparent in cross-hemispheric correlation strengths. If there is a reliable index of functional lateralization in resting-state correlation patterns, it will likely be necessary to examine not only the cross-hemispheric but also the intra-hemispheric correlations in a systematic manner. These measures would also need to be examined in conjunction with independent measures of the relevant behavioral functions, such as scores on a standardized language battery. All of that said, several of the brain regions with large FAD values appear to overlap with language-related cortex [7], suggesting a possible basis for those asymmetries. It may therefore be valuable for future studies to utilize landmark-based correspondence methods to jointly examine intra- and inter-hemispheric BOLD correlations, along with behavioral measures of cognitive functions that are believed to be lateralized.

## Supporting Information

Figure S1 **Diagram of the data processing steps used to calculate anatomical and functional correspondences by Landmark-Based Correspondence.** See the materials and methods section for the details.
(TIF)

Figure S2 **Functional correspondence for regions with high asymmetry.** (**A**) The seed regions with the highest FAD values (>100 mm ∼ upper 5%) are shown; small clusters with fewer than 50 nodes are excluded. In (**B**)–(**I**), lines connect individual seed vertices to their functional correspondences. Dorsal and ventral views of corresponding left and right seed vertices are shown. ROIs include (**B**) superior frontal sulcus; (**C**) inferior part of frontal sulcus, inferior part of precentral sulcus and gyrus; (**D**) supplementary motor area (see also Figure 5B); (**E**) anterior cingulate gyrus and sulcus, prefrontal cortex; (**F**) suborbital sulcus, rectus gyrus, medial olfactory gyrus, orbital gyrus, anterior circular sulcus of the insula; (**G**) planum polare of the superior temporal gyrus, anterior circular sulcus of the insula; (**H**) and (**I**) two clusters in temporal pole, middle temporal gyrus, inferior temporal gyrus (for **H**, see also Figure 5A).
(TIF)

Figure S3 **Correlation maps for regions with high asymmetry.** Correlation maps for seed ROIs (**B**)–(**I**) in Figure S2 with high FAD values are shown averaged over all corresponding vertices (for correlation thresholds, see colorbars to the right). Intra- and interhemispheric correlations (left and right seeds) are all rendered for ease of comparison on a common surface (left hemisphere) with lateral and medial views. Despite asymmetric patterns in the functional correspondences maps (Figure S2), the average correlation maps are largely symmetrical. This indicates that quantitative rather than qualitative differences drive the high FAD values, with the maximum correlation shifted to other “in-network” locations. **B**, **C**, **E**, **F**, **H**, and **I** all exhibit patterns reminiscent of language and/or “default” networks, whereas D corresponds mainly to primary and supplementary motor areas. In the cases of (**F**) and (**G**), the position of maximum correlation is shifted more for reasons of poor BOLD signal quality and larger noise at these seed locations.
(TIF)

Figure S4 **Hemispheric difference in temporal signal-to-noise (tSNR) ratios.** The functional asymmetric distances (FADs) can be biased by the tSNR difference across hemispheres at each seed pairs. Timeseries at seeds in right hemispheres were mapped on the left hemisphere following anatomical correspondences by landmark-based correspondence, and then the tSNRs of both hemispheres could be directly compared on the TT_N27 template surface by a Wilcoxon signed-rank test. There is no overlap between the high FAD seed pairs (black boundaries) and tSNR difference regions at the significance level uncorrected *p*<0.01 (filled in orange to red or light blue to blue colors).
(TIF)

Table S1 **AFCD and FAD for all Freesurfer ROIs used in Landmark Based Method.** The functional asymmetry distance (FAD) and anatomy-to-functional-correspondence distance (AFCD) is reported for each Freesurfer ROI used in implementing the Landmark-Based Correspondence method (see Figure 4A). Reported are the mean and standard deviation (SD) of FAD and AFCD within each ROI when calculated over vertices. ROIs are ordered by increasing FAD, from least to most asymmetric.
(DOC)
