# Correction: Quantifying Agreement between Anatomical and Functional Interhemispheric Correspondences in the Resting Brain

**DOI:** 10.1371/annotation/cb15c6af-2153-49a9-8330-45e40e6c296d

**Published:** 2013-05-24

**Authors:** Hang Joon Jo, Ziad S. Saad, Stephen J. Gotts, Alex Martin, Robert W. Cox

There is a sentence missing from the Acknowledgments:

The authors thank David A. Leopold and Brian E. Russ for helpful discussions. This research was supported by the NIMH and NINDS Intramural Research Programs of the NIH. 

