# Supplementary figures and images for: Quantifying Agreement between Anatomical and Functional Interhemispheric Correspondences in the Resting Brain
Source: PLoS One. 2012 Nov 8;7(11):e48847. doi: 10.1371/journal.pone.0048847 (PMC3493608; doi:10.1371/journal.pone.0048847)

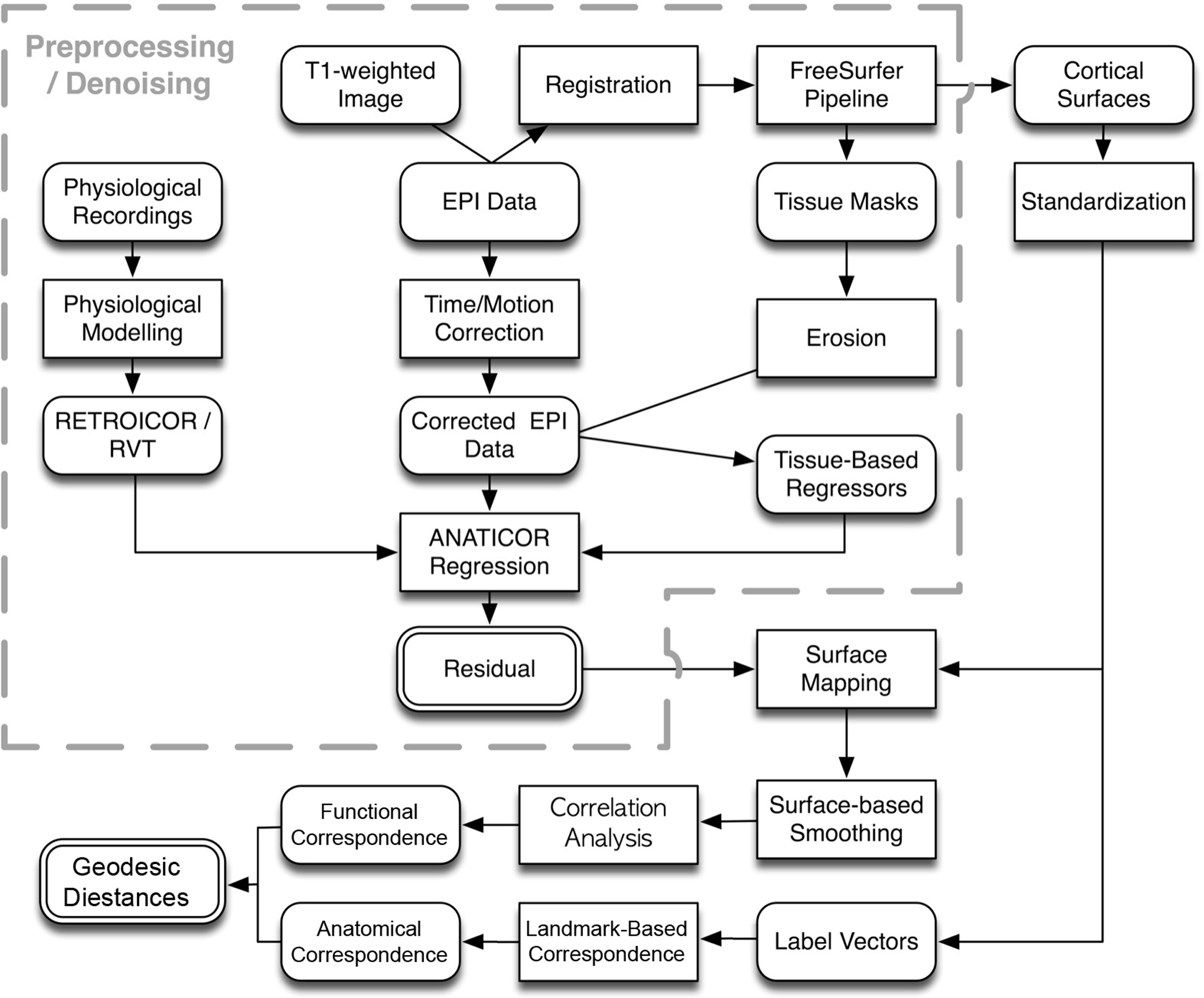

Supplement: Figure S1 — Diagram of the data processing steps used to calculate anatomical and functional correspondences by Landmark-Based Correspondence. See the materials and methods section for the details. (TIF) [file pone.0048847.s001.tif]

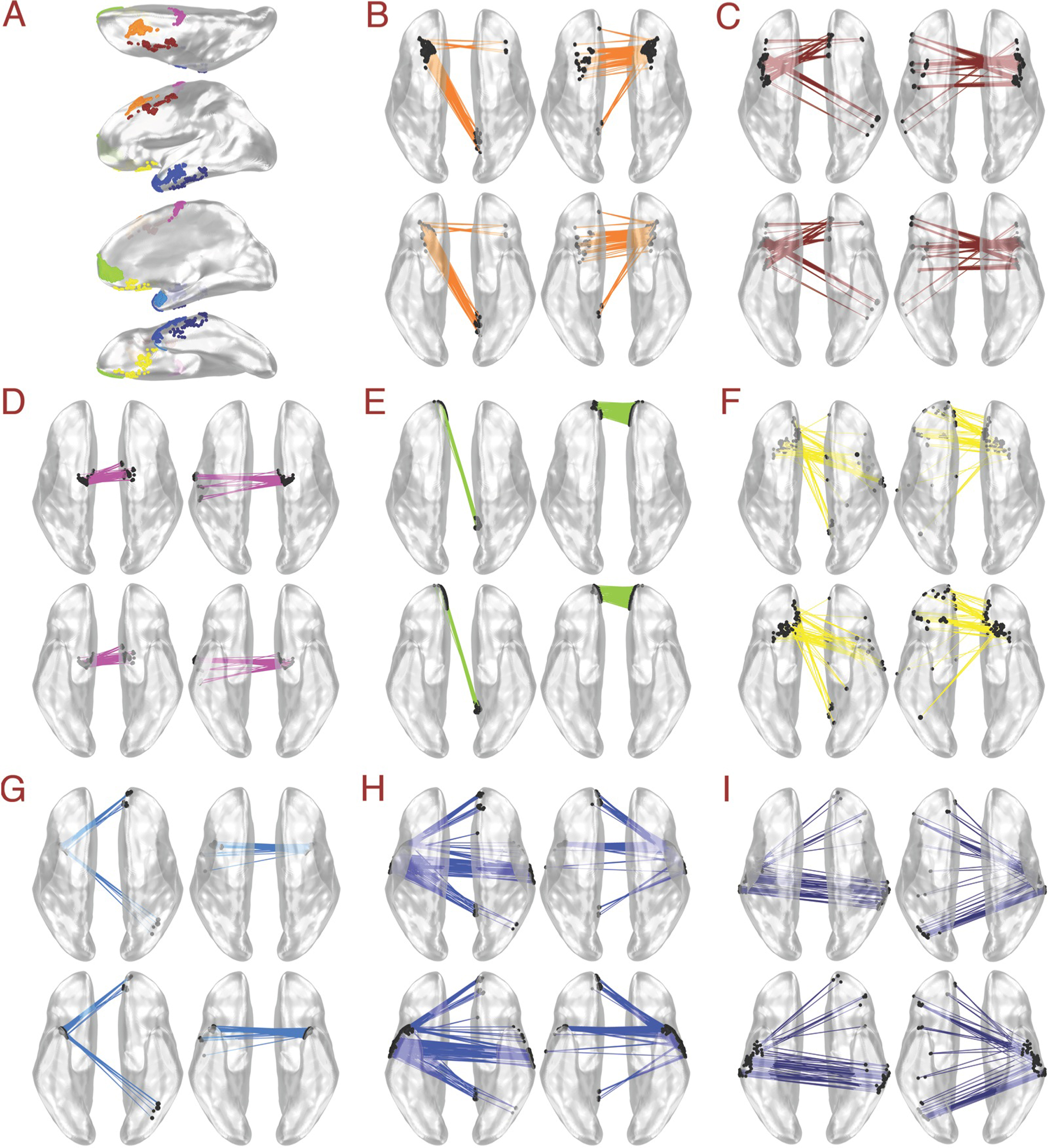

Supplement: Figure S2 — Functional correspondence for regions with high asymmetry. (A) The seed regions with the highest FAD values (>100 mm ∼ upper 5%) are shown; small clusters with fewer than 50 nodes are excluded. In (B)–(I), lines connect individual seed vertices to their functional correspondences. Dorsal and ventral views of corresponding left and right seed vertices are shown. ROIs include (B) superior frontal sulcus; (C) inferior part of frontal sulcus, inferior part of precentral sulcus and gyrus; (D) supplementary motor area (see also Figure 5B); (E) anterior cingulate gyrus and sulcus, prefrontal cortex; (F) suborbital sulcus, rectus gyrus, medial olfactory gyrus, orbital gyrus, anterior circular sulcus of the insula; (G) planum polare of the superior temporal gyrus, anterior circular sulcus of the insula; (H) and (I) two clusters in temporal pole, middle temporal gyrus, inferior temporal gyrus (for H, see also Figure 5A). (TIF) [file pone.0048847.s002.tif]

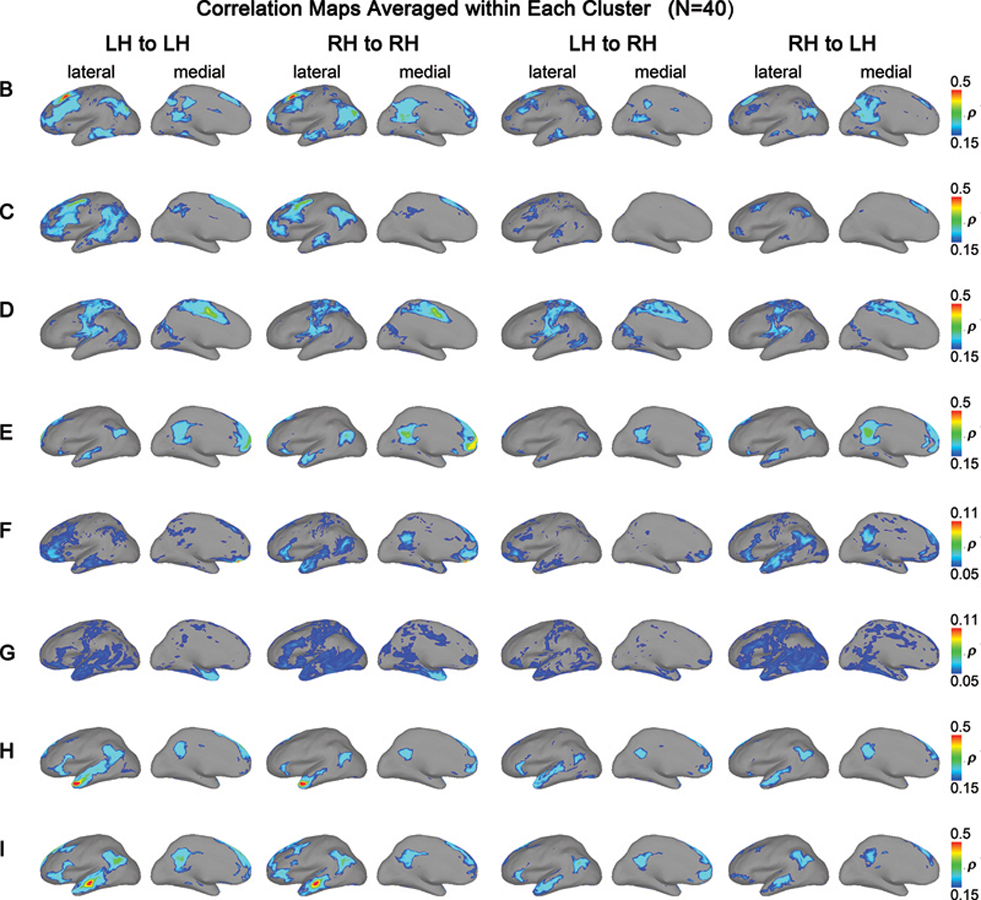

Supplement: Figure S3 — Correlation maps for regions with high asymmetry. Correlation maps for seed ROIs (B)–(I) in Figure S2 with high FAD values are shown averaged over all corresponding vertices (for correlation thresholds, see colorbars to the right). Intra- and interhemispheric correlations (left and right seeds) are all rendered for ease of comparison on a common surface (left hemisphere) with lateral and medial views. Despite asymmetric patterns in the functional correspondences maps (Figure S2), the average correlation maps are largely symmetrical. This indicates that quantitative rather than qualitative differences drive the high FAD values, with the maximum correlation shifted to other “in-network” locations. B, C, E, F, H, and I all exhibit patterns reminiscent of language and/or “default” networks, whereas D corresponds mainly to primary and supplementary motor areas. In the cases of (F) and (G), the position of maximum correlation is shifted more for reasons of poor BOLD signal quality and larger noise at these seed locations. (TIF) [file pone.0048847.s003.tif]

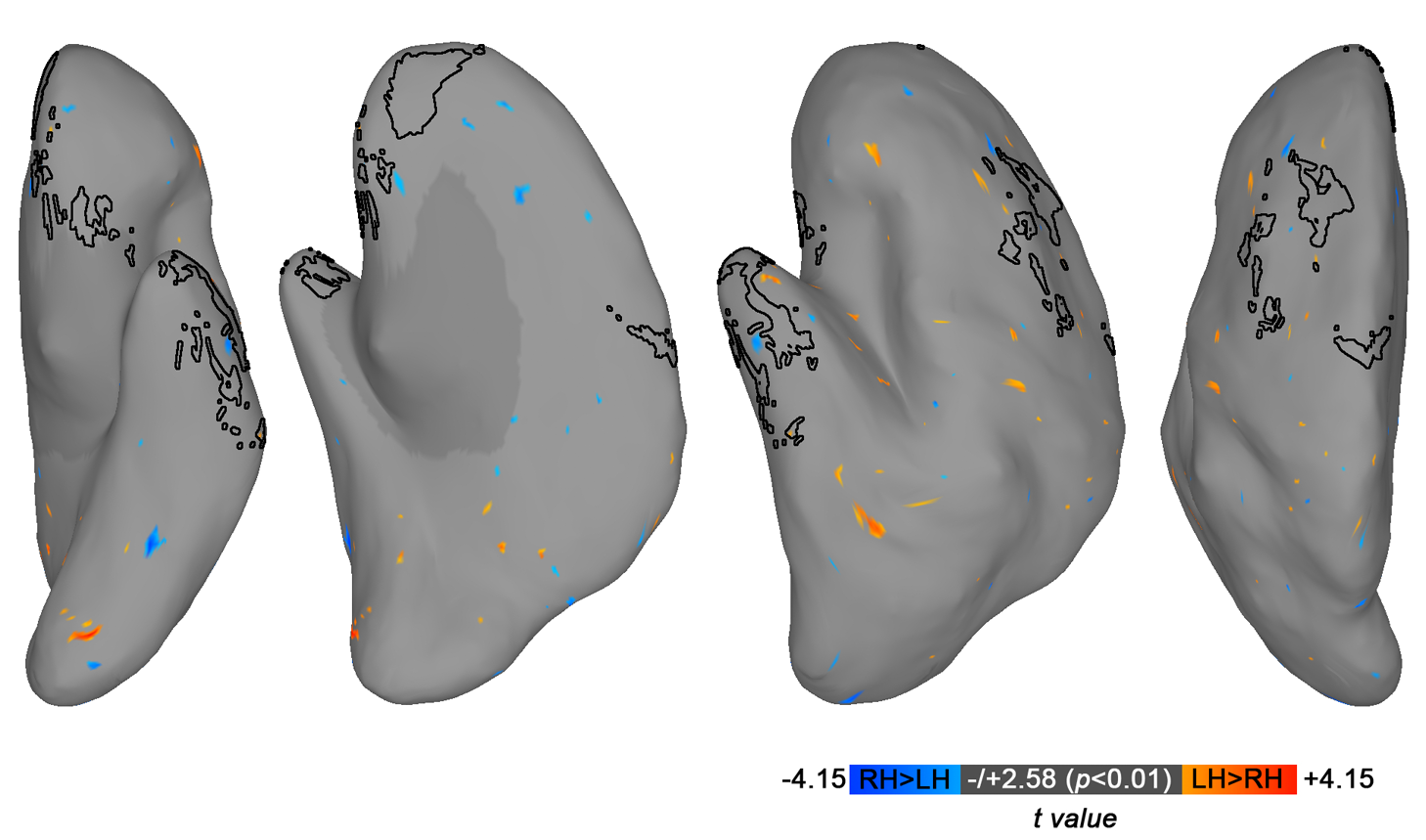

Supplement: Figure S4 — Hemispheric difference in temporal signal-to-noise (tSNR) ratios. The functional asymmetric distances (FADs) can be biased by the tSNR difference across hemispheres at each seed pairs. Timeseries at seeds in right hemispheres were mapped on the left hemisphere following anatomical correspondences by landmark-based correspondence, and then the tSNRs of both hemispheres could be directly compared on the TT_N27 template surface by a Wilcoxon signed-rank test. There is no overlap between the high FAD seed pairs (black boundaries) and tSNR difference regions at the significance level uncorrected p<0.01 (filled in orange to red or light blue to blue colors). (TIF) [file pone.0048847.s004.tif]
