# Supplementary material for: Quantifying Agreement between Anatomical and Functional Interhemispheric Correspondences in the Resting Brain
Source: PLoS One. 2012 Nov 8;7(11):e48847. doi: 10.1371/journal.pone.0048847 (PMC3493608; doi:10.1371/journal.pone.0048847)
Supplement: Table S1 — AFCD and FAD for all Freesurfer ROIs used in Landmark Based Method. The functional asymmetry distance (FAD) and anatomy-to-functional-correspondence distance (AFCD) is reported for each Freesurfer ROI used in implementing the Landmark-Based Correspondence method (see Figure 4A). Reported are the mean and standard deviation (SD) of FAD and AFCD within each ROI when calculated over vertices. ROIs are ordered by increasing FAD, from least to most asymmetric. (DOC) [file pone.0048847.s005.doc]

# Supporting Information

## Table S1. AFCD and FAD for all Freesurfer ROIs used in Landmark Based Method

|  |  |  |  | FAD (mm) | | |  | AFCD (mm) | | | | | | |  | Maximum *r*-value | | | | | | |
| --- | --- | --- | --- | --- | --- | --- | --- | --- | --- | --- | --- | --- | --- | --- | --- | --- | --- | --- | --- | --- | --- | --- |
| Rank |  | Region of interest |  |  |  |  |  | Left seeds | | |  | Right seeds | | |  | Left seeds | | |  | Right seeds | | |
|  |  |  |  | Mean |  | SD |  | Mean |  | SD |  | Mean |  | SD |  | Mean |  | SD |  | Mean |  | SD |
| 1 |  | Posterior-dorsal cingulate gyrus |  | 6.4 |  | 5.5 |  | 4.6 |  | 5.2 |  | 4.3 |  | 2.7 |  | 0.67 |  | 0.1 |  | 0.69 |  | 0.08 |
| 2 |  | Subcentral gyrus and sulcus |  | 8.5 |  | 6.6 |  | 5.4 |  | 3.9 |  | 7.3 |  | 6 |  | 0.67 |  | 0.07 |  | 0.68 |  | 0.08 |
| 3 |  | Pericallosal sulcus |  | 10 |  | 10.4 |  | 5.7 |  | 5.2 |  | 7.1 |  | 7.4 |  | 0.55 |  | 0.18 |  | 0.55 |  | 0.16 |
| 4 |  | Posterior-ventral cingulate gyrus |  | 11.1 |  | 9.5 |  | 9.7 |  | 4.7 |  | 5.5 |  | 7.8 |  | 0.71 |  | 0.15 |  | 0.58 |  | 0.21 |
| 5 |  | Transverse temporal sulcus |  | 12.2 |  | 4.3 |  | 8.3 |  | 3.3 |  | 11.6 |  | 4 |  | 0.62 |  | 0.08 |  | 0.75 |  | 0.03 |
| 6 |  | Anterior transverse temporal gyrus |  | 12.7 |  | 3.8 |  | 12.3 |  | 5.1 |  | 7.5 |  | 4.2 |  | 0.58 |  | 0.12 |  | 0.55 |  | 0.08 |
| 7 |  | Posterior ramus of the lateral ﬁssure |  | 13.5 |  | 8.9 |  | 9.5 |  | 5.7 |  | 8.5 |  | 7.3 |  | 0.64 |  | 0.07 |  | 0.62 |  | 0.05 |
| 8 |  | Planum temporale |  | 14.7 |  | 5.8 |  | 8 |  | 4.5 |  | 9.1 |  | 4.1 |  | 0.55 |  | 0.06 |  | 0.57 |  | 0.08 |
| 9 |  | Supramarginal gyrus |  | 15.9 |  | 10.8 |  | 8.2 |  | 4.8 |  | 9.8 |  | 9.5 |  | 0.59 |  | 0.09 |  | 0.57 |  | 0.09 |
| 10 |  | Superior circular sulcus of the insula |  | 16.2 |  | 13.1 |  | 11.9 |  | 12.3 |  | 9.3 |  | 11.4 |  | 0.55 |  | 0.08 |  | 0.55 |  | 0.06 |
| 11 |  | Calcarine sulcus |  | 18.1 |  | 7.6 |  | 12.3 |  | 5.3 |  | 9.4 |  | 4.3 |  | 0.75 |  | 0.1 |  | 0.77 |  | 0.09 |
| 12 |  | Vertical ramus of the anterior lateral ﬁssure |  | 19.1 |  | 8.4 |  | 12.3 |  | 4.9 |  | 14.5 |  | 5.5 |  | 0.52 |  | 0.06 |  | 0.44 |  | 0.04 |
| 13 |  | Subparietal sulcus |  | 20 |  | 6.5 |  | 15.5 |  | 9.1 |  | 14.1 |  | 6 |  | 0.55 |  | 0.08 |  | 0.64 |  | 0.09 |
| 14 |  | Parieto-occipital ﬁssure |  | 20.3 |  | 7.5 |  | 10.2 |  | 4.9 |  | 11.5 |  | 4.6 |  | 0.75 |  | 0.06 |  | 0.77 |  | 0.06 |
| 15 |  | Precuneus |  | 20.5 |  | 11.9 |  | 11 |  | 7.1 |  | 11.7 |  | 6.9 |  | 0.61 |  | 0.12 |  | 0.65 |  | 0.11 |
| 16 |  | Anterior circular sulcus of the insula |  | 21.2 |  | 24.8 |  | 15.9 |  | 24.2 |  | 7.2 |  | 4.8 |  | 0.47 |  | 0.1 |  | 0.49 |  | 0.07 |
| 17 |  | Lateral orbital sulcus |  | 21.6 |  | 21 |  | 13.6 |  | 3.7 |  | 11.3 |  | 23.4 |  | 0.43 |  | 0.04 |  | 0.38 |  | 0.04 |
| 18 |  | Opercular part of the inferior frontal gyrus |  | 21.7 |  | 23.6 |  | 10.1 |  | 11.4 |  | 17.6 |  | 22.9 |  | 0.46 |  | 0.09 |  | 0.45 |  | 0.1 |
| 19 |  | Triangular part of the inferior frontal gyrus |  | 22.9 |  | 6.7 |  | 15.8 |  | 7.5 |  | 16.8 |  | 8.1 |  | 0.43 |  | 0.06 |  | 0.39 |  | 0.04 |
| 20 |  | Fronto-marginal gyrus and sulcus |  | 23.6 |  | 7.6 |  | 13.9 |  | 7.7 |  | 12.7 |  | 4.4 |  | 0.37 |  | 0.04 |  | 0.36 |  | 0.03 |
| 21 |  | Orbital sulcus |  | 23.6 |  | 13.8 |  | 10.9 |  | 5.8 |  | 16.2 |  | 12.2 |  | 0.34 |  | 0.09 |  | 0.41 |  | 0.04 |
| 22 |  | Middle-posterior cingulate gyrus and sulcus |  | 23.9 |  | 38 |  | 8.6 |  | 4.8 |  | 19.5 |  | 40.4 |  | 0.48 |  | 0.07 |  | 0.49 |  | 0.07 |
| 23 |  | Lateral aspect of the superior temporal gyrus |  | 24.3 |  | 25 |  | 18.2 |  | 24.7 |  | 18.9 |  | 19.3 |  | 0.51 |  | 0.09 |  | 0.52 |  | 0.1 |
| 24 |  | Middle-anterior cingulate gyrus and sulcus |  | 24.6 |  | 40.6 |  | 8.9 |  | 4.9 |  | 20.8 |  | 39.4 |  | 0.48 |  | 0.05 |  | 0.5 |  | 0.04 |
| 25 |  | Paracentral lobule and sulcus |  | 24.7 |  | 9 |  | 12 |  | 6.3 |  | 15.1 |  | 9.4 |  | 0.55 |  | 0.04 |  | 0.55 |  | 0.04 |
| 26 |  | Lingual gyrus |  | 24.9 |  | 9.1 |  | 14.9 |  | 6.6 |  | 11.8 |  | 5.4 |  | 0.74 |  | 0.07 |  | 0.73 |  | 0.09 |
| 27 |  | Fusiform gyrus |  | 26.5 |  | 11.2 |  | 19.8 |  | 7.1 |  | 14.2 |  | 8.9 |  | 0.6 |  | 0.12 |  | 0.51 |  | 0.15 |
| 28 |  | Posterior transverse collateral sulcus |  | 27 |  | 2.8 |  | 13.9 |  | 2.7 |  | 15 |  | 2.4 |  | 0.73 |  | 0.04 |  | 0.71 |  | 0.03 |
| 29 |  | Cuneus |  | 27.6 |  | 5.8 |  | 15.1 |  | 5.1 |  | 13.7 |  | 3.9 |  | 0.76 |  | 0.07 |  | 0.78 |  | 0.06 |
| 30 |  | Inferior part of the precentral sulcus |  | 27.6 |  | 36.7 |  | 23.2 |  | 33 |  | 11.8 |  | 16.9 |  | 0.42 |  | 0.05 |  | 0.42 |  | 0.07 |
| 31 |  | Postcentral sulcus |  | 27.8 |  | 10.2 |  | 15.8 |  | 6.7 |  | 14.1 |  | 6.6 |  | 0.55 |  | 0.05 |  | 0.53 |  | 0.06 |
| 32 |  | Medial occipito-temporal and lingual sulcus |  | 28.4 |  | 9.8 |  | 16.7 |  | 8.4 |  | 14.3 |  | 5.5 |  | 0.61 |  | 0.13 |  | 0.6 |  | 0.1 |
| 33 |  | Central sulcus |  | 28.6 |  | 17.5 |  | 19.8 |  | 19 |  | 14.1 |  | 7.1 |  | 0.62 |  | 0.17 |  | 0.65 |  | 0.19 |
| 34 |  | Angular gyrus |  | 28.8 |  | 14.4 |  | 15.9 |  | 8.5 |  | 16.4 |  | 8 |  | 0.49 |  | 0.05 |  | 0.51 |  | 0.06 |
| 35 |  | Anterior transverse collateral sulcus |  | 29 |  | 23.3 |  | 19.7 |  | 13.2 |  | 20.3 |  | 23.1 |  | 0.34 |  | 0.09 |  | 0.29 |  | 0.05 |
| 36 |  | Intraparietal and transverse parietal sulcus |  | 29 |  | 11.9 |  | 17.7 |  | 8.1 |  | 14.8 |  | 6.1 |  | 0.53 |  | 0.04 |  | 0.54 |  | 0.08 |
| 37 |  | Marginal branch of the cingulate sulcus |  | 29.3 |  | 33.8 |  | 9.9 |  | 4.3 |  | 22.1 |  | 33.8 |  | 0.44 |  | 0.05 |  | 0.48 |  | 0.04 |
| 38 |  | Precentral gyrus |  | 29.4 |  | 18.5 |  | 17.5 |  | 16.8 |  | 16.5 |  | 11.9 |  | 0.47 |  | 0.06 |  | 0.51 |  | 0.07 |
| 39 |  | Orbital part of the inferior frontal gyrus |  | 29.5 |  | 24.3 |  | 17.6 |  | 27.2 |  | 16.4 |  | 8 |  | 0.43 |  | 0.03 |  | 0.42 |  | 0.03 |
| 40 |  | Inferior frontal sulcus |  | 29.8 |  | 32.5 |  | 24.8 |  | 31.7 |  | 14.4 |  | 16.7 |  | 0.38 |  | 0.03 |  | 0.38 |  | 0.03 |
| 41 |  | Superior parietal lobule |  | 30 |  | 12.3 |  | 15.4 |  | 9.2 |  | 16.6 |  | 6.8 |  | 0.55 |  | 0.04 |  | 0.57 |  | 0.06 |
| 42 |  | Postcentral gyrus |  | 30.1 |  | 18.5 |  | 18.7 |  | 17.7 |  | 14.9 |  | 5.7 |  | 0.63 |  | 0.17 |  | 0.57 |  | 0.1 |
| 43 |  | Long insular gyrus and central sulcus of the insula |  | 30.8 |  | 19.4 |  | 20.9 |  | 14.9 |  | 18.1 |  | 16.7 |  | 0.36 |  | 0.1 |  | 0.4 |  | 0.07 |
| 44 |  | Middle occipital and lunatus sulcus |  | 31.4 |  | 9.4 |  | 18.1 |  | 6.2 |  | 20.1 |  | 8.5 |  | 0.67 |  | 0.09 |  | 0.67 |  | 0.07 |
| 45 |  | Middle frontal sulcus |  | 31.9 |  | 26.8 |  | 14.7 |  | 9.4 |  | 21.2 |  | 25.6 |  | 0.4 |  | 0.04 |  | 0.38 |  | 0.05 |
| 46 |  | Superior temporal sulcus |  | 32.2 |  | 28.7 |  | 19.9 |  | 19.4 |  | 18.3 |  | 23 |  | 0.47 |  | 0.04 |  | 0.48 |  | 0.06 |
| 47 |  | Superior occipital and transverse occipital sulcus |  | 32.9 |  | 7.1 |  | 11.6 |  | 4.3 |  | 23.7 |  | 6.3 |  | 0.65 |  | 0.09 |  | 0.78 |  | 0.05 |
| 48 |  | Middle occipital gyrus |  | 33 |  | 13.6 |  | 17.2 |  | 9.2 |  | 19.1 |  | 8.5 |  | 0.6 |  | 0.09 |  | 0.63 |  | 0.08 |
| 49 |  | Horizontal ramus of the anterior lateral ﬁssure |  | 33.9 |  | 33.2 |  | 26.8 |  | 36.2 |  | 13.1 |  | 7 |  | 0.42 |  | 0.07 |  | 0.41 |  | 0.03 |
| 50 |  | Anterior occipital sulcus and preoccipital notch |  | 33.9 |  | 8.5 |  | 16.8 |  | 9.2 |  | 19.8 |  | 12.2 |  | 0.57 |  | 0.08 |  | 0.55 |  | 0.06 |
| 51 |  | Orbital gyrus |  | 35.2 |  | 28.1 |  | 23.6 |  | 25.1 |  | 22.9 |  | 19.3 |  | 0.32 |  | 0.11 |  | 0.36 |  | 0.07 |
| 52 |  | Transverse frontopolar gyrus and sulcus |  | 35.6 |  | 29.8 |  | 22.1 |  | 32.6 |  | 16.7 |  | 4.8 |  | 0.37 |  | 0.06 |  | 0.35 |  | 0.04 |
| 53 |  | Inferior circular sulcus of the insula |  | 35.7 |  | 23.1 |  | 19.4 |  | 18.5 |  | 23 |  | 16.3 |  | 0.34 |  | 0.1 |  | 0.37 |  | 0.09 |
| 54 |  | Superior part of the precentral sulcus |  | 36.6 |  | 21.7 |  | 21.5 |  | 19.9 |  | 18.6 |  | 16.3 |  | 0.42 |  | 0.04 |  | 0.46 |  | 0.04 |
| 55 |  | Occipital pole |  | 37.1 |  | 9.3 |  | 22.4 |  | 7.7 |  | 17.3 |  | 5.9 |  | 0.62 |  | 0.08 |  | 0.62 |  | 0.05 |
| 56 |  | Inferior occipital gyrus and sulcus |  | 38.3 |  | 17.6 |  | 18.4 |  | 10.2 |  | 27.4 |  | 22.8 |  | 0.67 |  | 0.04 |  | 0.66 |  | 0.07 |
| 57 |  | Superior occipital gyrus |  | 38.4 |  | 7.1 |  | 20.7 |  | 8.2 |  | 19.8 |  | 9.6 |  | 0.74 |  | 0.1 |  | 0.77 |  | 0.08 |
| 58 |  | Anterior cingulate gyrus and sulcus |  | 39.1 |  | 40.8 |  | 31.9 |  | 41.5 |  | 12.2 |  | 4.5 |  | 0.43 |  | 0.1 |  | 0.45 |  | 0.06 |
| 59 |  | Superior frontal gyrus |  | 39.4 |  | 35.3 |  | 20.7 |  | 29.1 |  | 21.6 |  | 26.6 |  | 0.48 |  | 0.06 |  | 0.48 |  | 0.06 |
| 60 |  | Middle frontal gyrus |  | 42 |  | 43.4 |  | 31.8 |  | 35.9 |  | 30 |  | 38 |  | 0.39 |  | 0.04 |  | 0.38 |  | 0.04 |
| 61 |  | Lateral occipito-temporal sulcus |  | 43.2 |  | 17.8 |  | 28.7 |  | 10.4 |  | 22.3 |  | 29.9 |  | 0.55 |  | 0.06 |  | 0.45 |  | 0.04 |
| 62 |  | Straight gyrus, rectus gyrus |  | 48.4 |  | 37.3 |  | 40.7 |  | 34.6 |  | 25.9 |  | 21.4 |  | 0.22 |  | 0.08 |  | 0.25 |  | 0.08 |
| 63 |  | Short insular gyrus |  | 49.7 |  | 32.6 |  | 39.8 |  | 37.8 |  | 23.2 |  | 20.4 |  | 0.35 |  | 0.07 |  | 0.44 |  | 0.07 |
| 64 |  | Temporal pole |  | 49.8 |  | 47.7 |  | 48.5 |  | 52 |  | 20.5 |  | 15.7 |  | 0.25 |  | 0.05 |  | 0.25 |  | 0.08 |
| 65 |  | Inferior temporal gyrus |  | 52.7 |  | 45.7 |  | 35.9 |  | 36.6 |  | 37.2 |  | 43.6 |  | 0.37 |  | 0.14 |  | 0.33 |  | 0.12 |
| 66 |  | Subcallosal area, subcallosal gyrus |  | 56.4 |  | 36.3 |  | 48 |  | 34.2 |  | 15.9 |  | 17.3 |  | 0.21 |  | 0.04 |  | 0.26 |  | 0.05 |
| 67 |  | Intermedius primus sulcus |  | 56.4 |  | 10.3 |  | 21.9 |  | 5.1 |  | 38.6 |  | 11.8 |  | 0.48 |  | 0.04 |  | 0.45 |  | 0.02 |
| 68 |  | Middle temporal gyrus |  | 59.1 |  | 55.5 |  | 31.5 |  | 46.9 |  | 49.8 |  | 56.4 |  | 0.4 |  | 0.05 |  | 0.41 |  | 0.05 |
| 69 |  | Parahippocampal gyrus |  | 60.6 |  | 33.6 |  | 28.5 |  | 18.7 |  | 42.6 |  | 29.9 |  | 0.34 |  | 0.14 |  | 0.22 |  | 0.04 |
| 70 |  | Superior frontal sulcus |  | 65.9 |  | 55.1 |  | 53.4 |  | 56.2 |  | 35.6 |  | 51 |  | 0.39 |  | 0.03 |  | 0.37 |  | 0.03 |
| 71 |  | Suborbital sulcus |  | 70.1 |  | 49.1 |  | 63.2 |  | 46.7 |  | 17 |  | 12 |  | 0.29 |  | 0.11 |  | 0.33 |  | 0.12 |
| 72 |  | Medial orbital sulcus |  | 76.2 |  | 50.2 |  | 65.5 |  | 47.7 |  | 32.6 |  | 20.4 |  | 0.2 |  | 0.05 |  | 0.24 |  | 0.02 |
| 73 |  | Planum polare of the superior temporal gyrus |  | 83.7 |  | 44.7 |  | 70.8 |  | 44.2 |  | 25.9 |  | 22.2 |  | 0.29 |  | 0.1 |  | 0.24 |  | 0.06 |
| 74 |  | Inferior temporal sulcus |  | 86.8 |  | 67.8 |  | 54.6 |  | 60 |  | 76.9 |  | 70.2 |  | 0.35 |  | 0.09 |  | 0.32 |  | 0.1 |

The functional asymmetry distance (FAD) and anatomy-to-functional-correspondence distance (AFCD) is reported for each Freesurfer ROI used in implementing the Landmark-Based Correspondence method (see Figure 4A). Reported are the mean and standard deviation (SD) of FAD and AFCD within each ROI when calculated over vertices. ROIs are ordered by increasing FAD, from least to most asymmetric.

## Figure S1. Diagram of the data processing steps used to calculate anatomical and functional correspondences by Landmark-Based Correspondence.


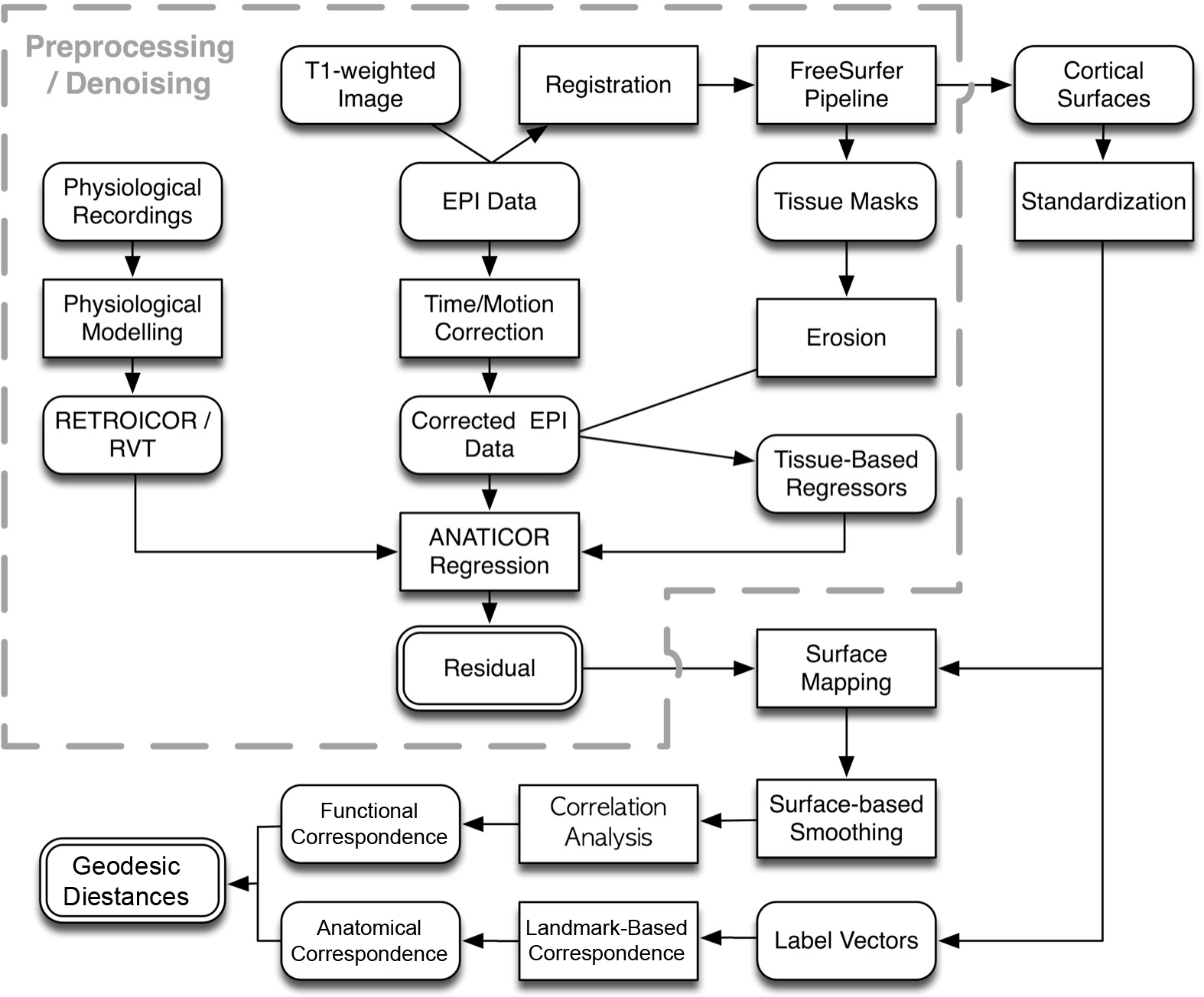


## Figure S2. Functional correspondence for regions with high asymmetry

##
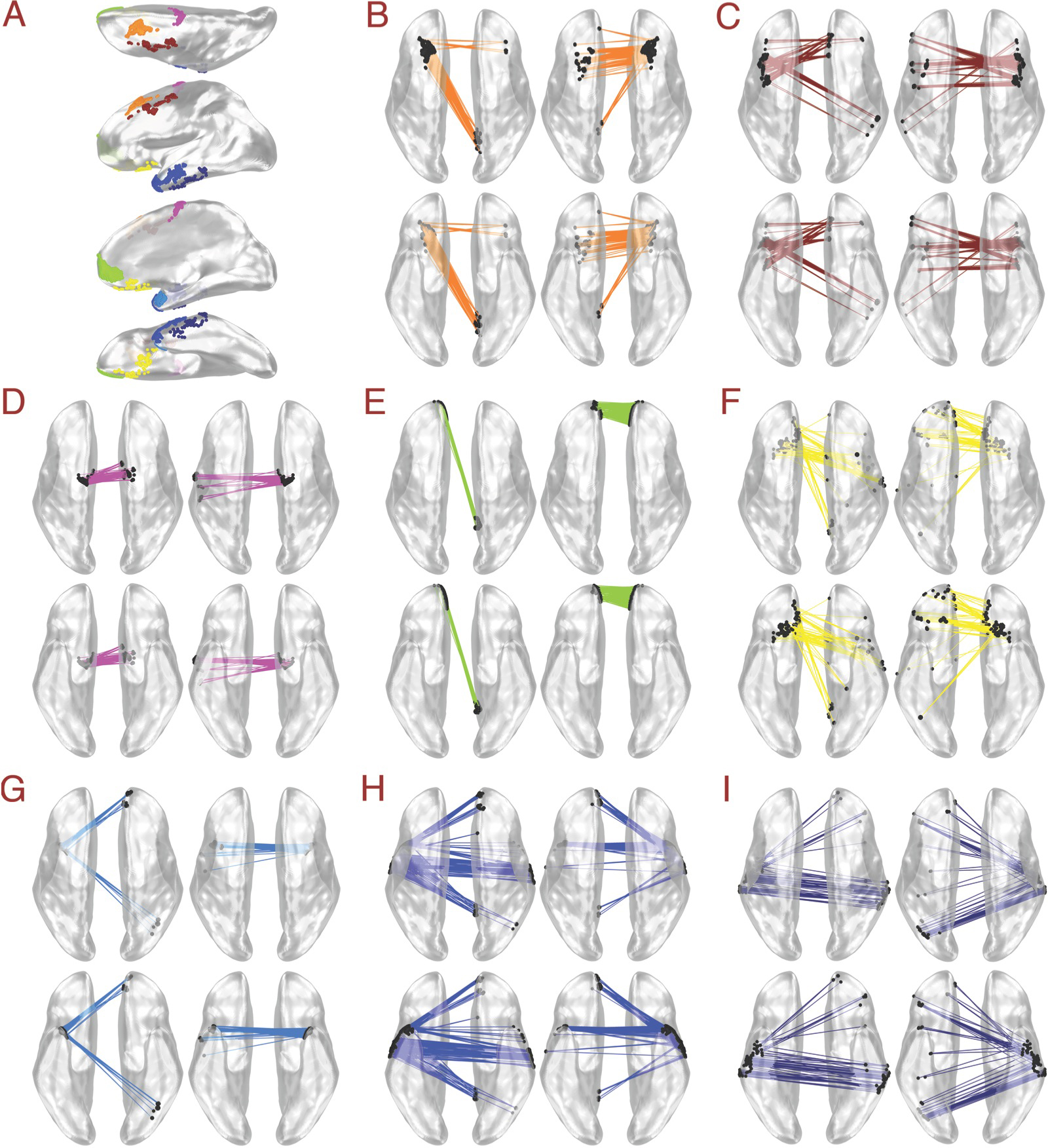


(**A**) The seed regions with the highest FAD values (> 100 mm ~ upper 5 %) are shown; small clusters with fewer than 50 nodes are excluded. In (**B**)-(**I**), lines connect individual seed vertices to their functional correspondences. Dorsal and ventral views of corresponding left and right seed vertices are shown. ROIs include (**B**) superior frontal sulcus; (**C**) inferior part of frontal sulcus, inferior part of precentral sulcus and gyrus; (**D**) supplementary motor area (see also Figure 5B); (**E**) anterior cingulate gyrus and sulcus, prefrontal cortex; (**F**) suborbital sulcus, rectus gyrus, medial olfactory gyrus, orbital gyrus, anterior circular sulcus of the insula; (**G**) planum polare of the superior temporal gyrus, anterior circular sulcus of the insula; (**H**) and (**I**) two clusters in temporal pole, middle temporal gyrus, inferior temporal gyrus (for **H**, see also Figure 5A).

## Figure S3. Correlation maps for regions with high asymmetry


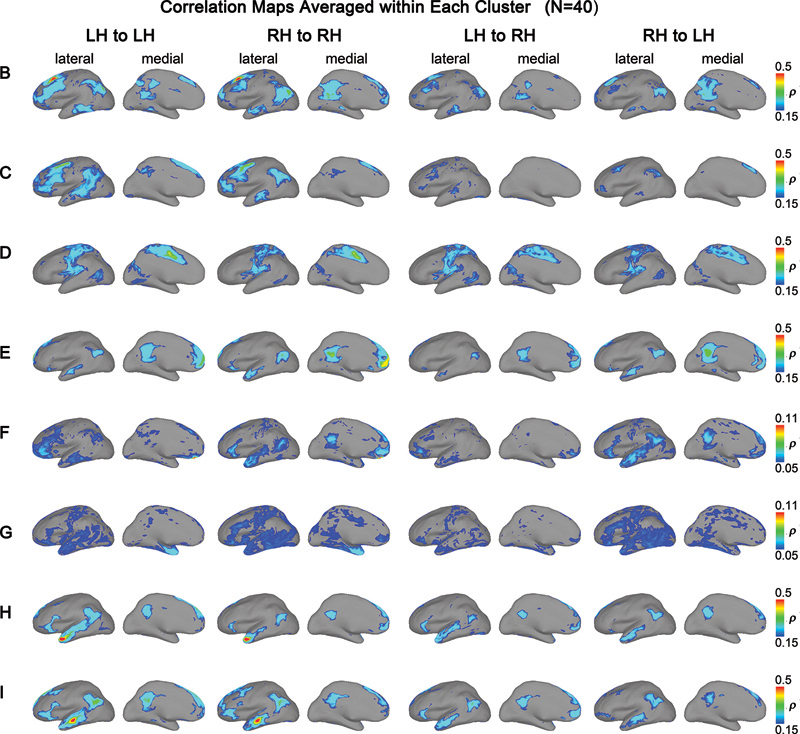


Correlation maps for seed ROIs (**B**)-(**I**) in Figure S2 with high FAD values are shown averaged over all corresponding vertices (for correlation thresholds, see colorbars to the right). Intra- and interhemispheric correlations (left and right seeds) are all rendered for ease of comparison on a common surface (left hemisphere) with lateral and medial views. Despite asymmetric patterns in the functional correspondences maps (Figure S2), the average correlation maps are largely symmetrical. This indicates that quantitative rather than qualitative differences drive the high FAD values, with the maximum correlation shifted to other "in-network" locations. **B**, **C**, **E**, **F**, **H**, and **I** all exhibit patterns reminiscent of language and/or "default" networks, whereas D corresponds mainly to primary and supplementary motor areas. In the cases of (**F**) and (**G**), the position of maximum correlation is shifted more for reasons of poor BOLD signal quality and larger noise at these seed locations.

## Figure S4. Hemispheric difference in temporal signal-to-noise (tSNR) ratios


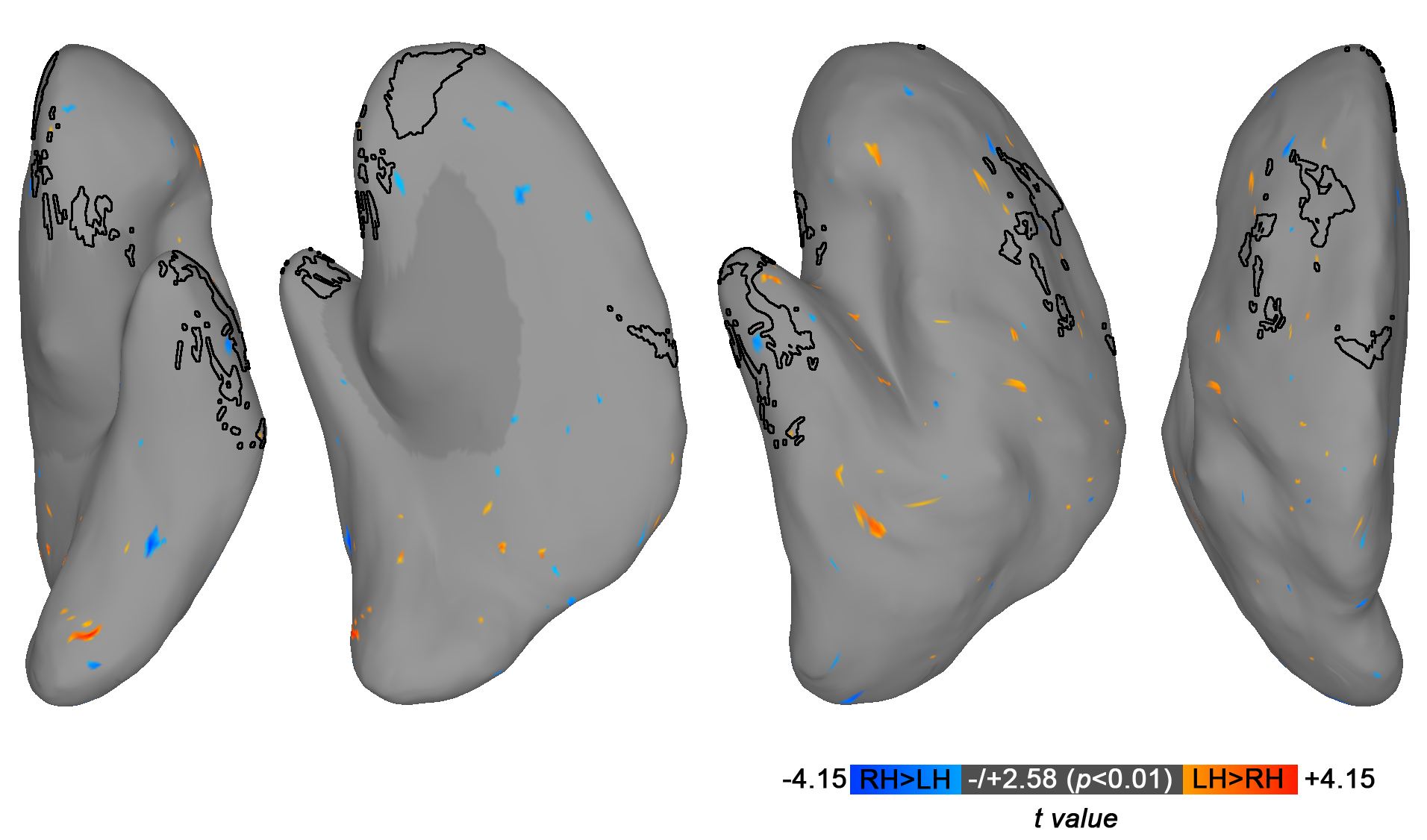


The functional asymmetric distances (FADs) can be biased by the tSNR difference across hemispheres at each seed pairs. Timeseries at seeds in right hemispheres were mapped on the left hemisphere following anatomical correspondences by landmark-based correspondence, and then the tSNRs of both hemispheres could be directly compared on the TT_N27 template surface by a Wilcoxon signed-rank test. There is no overlap between the high FAD seed pairs (black boundaries) and tSNR difference regions at the significance level uncorrected p<0.01 (filled in orange to red or light blue to blue colors).
